# 

**DOI:** 10.1192/bjb.2025.11

**Published:** 2026-02

**Authors:** Rachel Gibbons

**Affiliations:** Independent researcher, London, UK. Email: rachelgibbons@me.com

**Figure:**
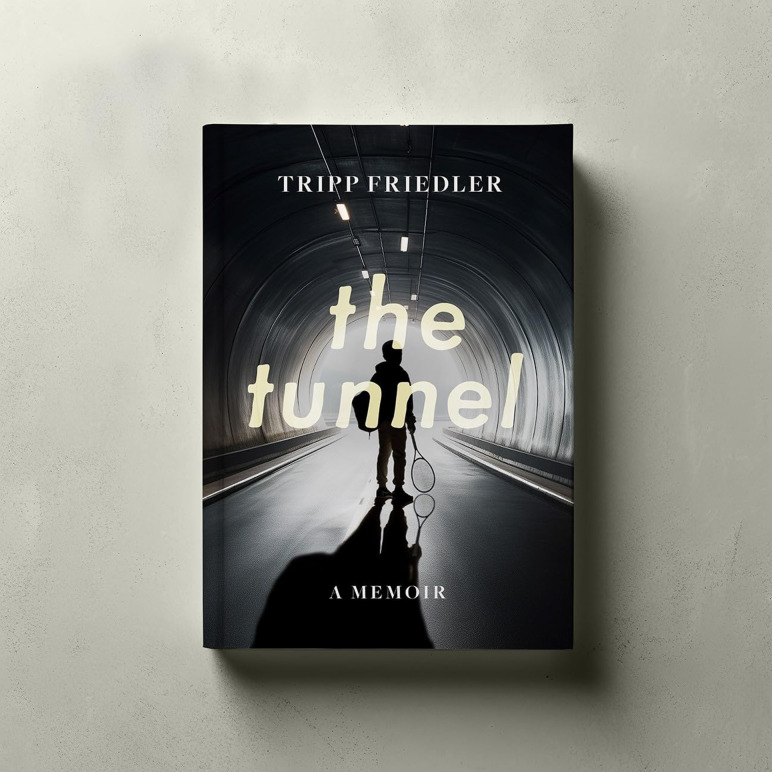

*The Tunnel: A Memoir* by Tripp Friedler is a powerful account of love, loss and the consuming nature of serious mental illness, told through the eyes of a grieving father. It recounts his son Henry's struggles with bipolar disorder, repeated hospitalisations and ultimate death by suicide. The memoir offers rare insight into the devastating impact of severe mental illness on families, capturing their entrapment in cycles of hope and despair. The narrative explores profound layers of grief: the loss of the child you hoped for, the family dynamic you imagined, the depletion of resources and the impact on siblings. Friedler's account raises haunting questions about the nature and trajectory of mental illness. Were there earlier signs of Henry's struggles that went unnoticed, or were they simply absent? Is death by suicide solely a result of mental illness, or can mental illness sometimes act as a fragile form of protection? Henry's death, occurring just as he began to show signs of recovery, reflects a pattern seen in many others.

Friedler explores the destructive power of mental illness, capturing the emotional, spiritual and financial toll of caregiving in a culture that often criminalises rather than supports the unwell. At one point, treatment costs exceeded $100 000 for just a few months’ care. The memoir highlights the unique brutality of US systems, from inadequate school and college accommodation for mental health difficulties to the pervasive availability of firearms. The book is both gripping and emotionally intense, drawing readers into the father's relentless journey. A key strength is its portrayal of his gradual transformation, from attempting to ‘fix’ Henry through sporting and academic achievements to accepting the harsh reality of his condition. However, the single, focused perspective is also a limitation. The absence of other voices, especially Henry's, creates a void – an inevitable reality after suicide.

This book highlights how suicide is both deeply feared and inherently unpredictable, even in high-risk individuals. It captures the difficulty in understanding severe mental illness and suicide, and the unfair self-blame often felt by the bereaved. The father's narrative echoes common grief questions: ‘What did I miss? What could I have done differently?’ Most importantly, this book forces us to confront the complexity of compassion, challenging readers to find understanding for both Henry and his father. It reminds us that some losses, no matter the support structures, cannot be avoided – only survived.

I strongly recommend *The Tunnel: A Memoir*. Told with remarkable candour by a father in the early stages of grief, this is a rare and valuable story. With suicide the leading cause of death for men under 50 in the UK, this account is vital. I hope Mr Friedler found some solace in sharing his family's story. *The Tunnel* is a poignant memorial to Henry.

